# Cardiometabolic risk in a population of older adults with multiple co-morbidities in rural south africa: the HAALSI (Health and Aging in Africa: longitudinal studies of INDEPTH communities) study

**DOI:** 10.1186/s12889-017-4117-y

**Published:** 2017-02-17

**Authors:** Thomas A. Gaziano, Shafika Abrahams-Gessel, F. Xavier Gomez-Olive, Alisha Wade, Nigel J. Crowther, Sartaj Alam, Jennifer Manne-Goehler, Chodziwadziwa W Kabudula, Ryan Wagner, Julia Rohr, Livia Montana, Kathleen Kahn, Till W. Bärnighausen, Lisa F. Berkman, Stephen Tollman

**Affiliations:** 1 Department of Cardiovascular Medicine, Brigham & Women’s Hospital, 75 Francis Street, Boston, MA 02115 USA; 2 Center for Health Decision Science, Harvard T.H. Chan School of Public Health, 718 Huntington Ave., Boston, MA 02115 USA; 3 Harvard Center for Population and Development Studies, Harvard University, 9 Bow Street, Cambridge, MA 02138 USA; 4 Medical Research Council/Wits Rural Public Health and Health Transitions Research Unit, School of Public Health, Faculty of Health Sciences, University of the Witwatersrand, 27 St. Andrew’s Road, Johannesburg, Parktown 2193 South Africa; 5 INDEPTH Network, Accra, Ghana; 6 Africa Wits-INDEPTH Genomic Studies of Cardiovascular Disease, University of the Witwatersrand, Johannesburg, South Africa; 7 School of Public Health, University of the Witwatersrand, Johannesburg, South Africa; 8 National Health Laboratory Service and Department of Chemical Pathology, Faculty of Health Sciences, University of the Witwatersrand, 7 York Rd., Johannesburg, Parktown 2193 South Africa; 9 Department of Health Care Policy, Harvard Medical School, 180 Longwood Avenue, Boston, MA 02115 USA; 10 Department of Medicine, Beth Israel Deaconess Medical Center, Harvard Medical School, 330 Brookline Ave, Boston, MA 02215 USA; 11 Department of Global Health & Population, Harvard T.H. Chan School of Public Health, Boston, MA USA; 12 Department of Population Health, London School of Hygiene and Tropical Medicine, London, UK; 13 Umeå Centre for Global Health Research, Division of Epidemiology and Global Health, Department of Public Health and Clinical Medicine, Umeå University, Umea, Sweden; 14 Africa Health Research Institute (AHRI), Mtubatuba, South Africa; 15 Institute of Public Health, Faculty of Medicine, University of Heidelberg, INF 130.3, Heidelberg, Baden-Württemberg 69120 Germany

**Keywords:** Cardiovascular, Cardiometabolic, HIV, Aging, Antiretrovirals, Hypertension, Diabetes

## Abstract

**Background:**

A consequence of the widespread uptake of anti-retroviral therapy (ART) is that the older South African population will experience an increase in life expectancy, increasing their risk for cardiometabolic diseases (CMD), and its risk factors. The long-term interactions between HIV infection, treatment, and CMD remain to be elucidated in the African population. The HAALSI cohort was established to investigate the impact of these interactions on CMD morbidity and mortality among middle-aged and older adults.

**Methods:**

We recruited randomly selected adults aged 40 or older residing in the rural Agincourt sub-district in Mpumalanga Province. In-person interviews were conducted to collect baseline household and socioeconomic data, self-reported health, anthropometric measures, blood pressure, high-sensitivity C-reactive protein (hsCRP), HbA1c, HIV-status, and point-of-care glucose and lipid levels.

**Results:**

Five thousand fifty nine persons (46.4% male) were enrolled with a mean age of 61.7 ± 13.06 years. Waist-to-hip ratio was high for men and women (0.92 ± 0.08 vs. 0.89 ± 0.08), with 70% of women and 44% of men being overweight or obese. Blood pressure was similar for men and women with a combined hypertension prevalence of 58.4% and statistically significant increases were observed with increasing age. High total cholesterol prevalence in women was twice that observed for men (8.5 vs. 4.1%). The prevalence of self-reported CMD conditions was higher among women, except for myocardial infarction, and women had a statistically significantly higher prevalence of angina (10.82 vs. 6.97%) using Rose Criteria. The HIV^−^ persons were significantly more likely to have hypertension, diabetes, or be overweight or obese than HIV^+^ persons. Approximately 56% of the cohort had at least 2 measured or self-reported clinical co-morbidities, with HIV^+^ persons having a consistently lower prevalence of co-morbidities compared to those without HIV. Absolute 10-year risk cardiovascular risk scores ranged from 7.7–9.7% for women and from 12.5–15.3% for men, depending on the risk score equations used.

**Conclusions:**

This cohort has high CMD risk based on both traditional risk factors and novel markers like hsCRP. Longitudinal follow-up of the cohort will allow us to determine the long-term impact of increased lifespan in a population with both high HIV infection and CMD risk.

## Background

At a global level, growth in the proportion of adults aged 65 and older is outpacing the proportion of younger adults for the first time [1]. As a result, chronic diseases, including cardiometabolic diseases (CMD), have become a major burden of disease and related mortality in low-resource settings, including sub-Saharan Africa (SSA) [2–4]. From the late 1990s to 2011, non-communicable disease mortality increased steadily [3] in adults 50 years and above, due largely to an increase in a number of risk factors such as hypertension, increased smoking prevalence, dietary changes and obesity [5]. These risks factors have been followed by increases in symptomatic cardiovascular conditions such as stroke, ischemic heart disease and diabetes, for which health policymakers have yet to evolve an effective response.

These trends are prominent despite the pervasive effects of human immunodeficiency virus/acquired immune deficiency syndrome (HIV/AIDS). Further, it is increasingly recognized that human populations in sub-Saharan Africa, but especially in South Africa, in spite of a previous profound drop in life expectancy, are now aging rapidly. This demographic transition is the result of falling mortality rates, due in part to widespread uptake of anti-retroviral therapy (ART), coupled with marked decreases in fertility in recent decades [1, 6, 7]. National leaders, health and social systems, and the accompanying policy processes—still contending with an “unfinished agenda” of widespread infection and excess maternal, infant and childhood illness—are poorly prepared for the scale and speed of the demographic change that is underway [8].

The transition is complex, with a non-linear trajectory, reflected in the ‘collision’ between non-communicable and infectious disease [9]. Widespread uptake of ART for HIV has led to a profound improvement in mortality trends at all ages [10]. While ART extends the lifespan of people living with HIV/AIDS (PLWHA), it does not come without costs. Compared to PLWHA who were not on ART, those on ART have been shown to have an increased prevalence of dyslipidemia [6, 11, 12]. Biomarkers for inflammation, e.g. C-reactive protein (CRP), have also been identified as potential useful markers for assessing increased risk of CMD in HIV-infected persons, though the evidence is mixed [7, 13–15]. The long-term effect of the complex interaction between epidemic infectious disease and CMD in these environments is simply not known and will require prospective follow-up of population-based cohorts to answer this question.

The shifting factors that have led to the increase in CMD in South Africa are certainly exerting pressure to act, but public sector policymakers and programmers face a critical barrier: a lack of population-based data with which to understand the multiple effects and determinants of the ongoing transition, specifically with regard to CMD. In 2015 as part of the Health and Aging in Africa: A Longitudinal Study of an INDEPTH Community (HAALSI) in South Africa, we completed the baseline wave of data collection in a cohort of 5,059 men and women ≥40 years of age drawn from the Agincourt Health and socio-Demographic Surveillance System (HDSS) which covers the Agincourt sub-district of Mpumalanga Province in a border region of rural South Africa adjacent to southern Mozambique [16]. The overall objective of HAALSI with regard to CMD is to understand the major forces shaping the trajectory of the prevalence, incidence, and risk factors of CMD in this cohort of 5,059 individuals. In this particular study, we sought to assess the prevalence of CMD risk factors and symptomatic CMDs and to measure the 10-year CMD risk in a large rural South African population. Second, we sought to compare these variables across genders and HIV status.

## Methods

The HAALSI study is based in the Agincourt HDSS site, a sub-district of rural Mpumalanga Province comprising some 116,000 people living in 19,000 households and 31 villages in an area of ~450 km^2^. An annual census update, conducted by experienced local field staff, provides up-to-date denominator data on the full population with systematic recording of all vital events (deaths, births, in- / out-migrations).

### Eligibility

All adults aged 40 years and older as of July 1, 2014, who had permanently resided in the Agincourt sub-district of Mpumalanga Province for at least one year prior to the 2013 census update, were eligible.

### Sampling and sample size

A total of 6,281 persons were selected for recruitment. To maximize the linkages with previous HIV and NCD studies in Agincourt, those who participated in earlier studies and met the eligibility criteria were selected with 100 percent probability. The remainder of the sample was selected randomly from the 2013 census HDSS database, stratifying on sex in order to achieve equal numbers of women and men.

### Recruitment

Prior to the survey, the HAALSI study was introduced to community members across the study villages, and discussed in-depth with a representative Community Advisory Group. This facilitated community review of study objectives and contributed to the effective response achieved. The entire population identifies itself as “African”.

Between November 2014 and November 2015 all selected individuals were visited at home by a supervised local field worker who briefly described the study in the local language (Shangaan), and requested permission to explain the relevant informed consent forms. A signed consent form was obtained from those who agreed to participate. Field workers also signed and dated the informed consent form.

### Data collection

Interviews lasting 2–3 hours were conducted at participants’ households. The visit included a questionnaire in two parts: household and socioeconomic data then individual interview data, which included anthropometric, physical and cognitive function assessments plus blood sample collection in the form of a capillary blood sample or “dried blood spots” (DBS). Data were captured on laptop computers using a Computer Assisted Personal Interview (CAPI) program. All self-reported conditions were captured using the question “Have you ever been told by a doctor, nurse, or other healthcare worker that you have (condition)?” Physical data collected included blood pressure (Omron M6W® automated cuff), weight (Genesis Growth Management Electronic Scale®); height using a height sensor with infrared measurement; waist and hip circumferences with a flexible tape measure (SECA®). The DBS were analyzed for high-sensitivity C-reactive protein (hsCRP), HIV-status, viral load, nucleoside reverse transcriptase inhibitors among the HIV^+^ (1–3 drops), and presence of the anti-retroviral drugs (ART) emtricitabine or lamivudine for persons with a positive HIV antibody test. The Division of Clinical Pharmacology Laboratory at the University of Cape Town (UCT), South Africa performed the testing for the presence of ART. Additional blood samples were used in point-of-care machines to measure glucose (Caresens^©^ N Monitor), and individual lipid levels (Cardiochek^©^ PA Silver version. Three blood pressure readings (systolic and diastolic) were obtained with 2 minutes between each reading.

### Assessment of CVD risk factors and HIV status and diagnosis of CMDs

Age stratification follows the South African National Health and Nutrition Examination Survey (SANHANES) categorization [17]. Body Mass Index (BMI; kg/m^2^) categorization uses World Health Organization (WHO) obesity cutoffs [18] and waist-to-hip ratios (WHR) were considered high if > 0.90 for men, and > 0.85 for women [19]. The mean blood pressure was calculated using the average between the second and third readings [20]. Hypertension was defined as a mean systolic blood pressure ≥140 mmHg or mean diastolic blood pressure ≥ 90 mmHg or controlled blood pressure with self-reported use of taking hypertensive medication [21]. Diabetes was defined as a self-reported history, or a fasting blood glucose level (FBG) ≥ 7.0 mmol/L, or a random blood glucose level (RBG) ≥ 11.1 mmol/L or reported use of medication [22]. A FBG required no caloric intake for at least 8 hours prior to the collection of the blood sample. Dyslipidemia was defined as a self-reported history, or measured total cholesterol level ≥ 6.21 mmol/L, or low-density lipoprotein (LDL) > 4.1 mmol/L, or high-density lipoprotein (HDL) < 1.19 mmol/L, or triglyceride > 2.2.5 mmol/L or taking medication for the condition [23, 24].

The hsCRP was categorized as low (<1 mg/L), intermediate (1–3 mg/L), or high (>3 mg/L) [25, 26]. Absolute risk of suffering a cardiac-related event or death (CVD risk) over ten years following the baseline interview was calculated using Pooled Risk Score Equations (ASCVD) [27] and Framingham Score [28], while risk of CVD death was calculated using the Harvard Risk Score [29]. These three risk scores use different combinations of age, sex, smoking status, systolic blood pressure, reported diabetes, and either cholesterol values or body-mass index to predict risk. Angina was defined as self-report, or using a validated subset of questions from the Rose Angina Questionnaire relating to chest pain, exertion, and symptom resolution [30–32]. Self-reported current medication use for hypertension, diabetes, and dyslipidemia was also obtained during the interview. For prevalence of HIV we used the blood results from a protocol of sequential assays. Specifically, the Vironostika Uniform 11 (Biomeriuex, France) screening assay was used to detect the presence of the virus; and in samples that tested positive, the Roche Elecys (USA) assay (confirmatory) was used to determine the viral load on DBS. Contradictory results were resolved using the Siemens Centaur XP (USA) immunoassay, in accordance with World Health Organization (WHO) guidelines [33, 34]. HIV^+^ persons who tested positive for the presence of ART medications were deemed compliant with ARV treatment. All analyses were conducted using SAS ® V9.4 and STATA ® V14 software.

## Results

### General population

Out of the 5,890 persons identified for recruitment who were alive and residing in the study area, 85.9% (5,059) agreed to be interviewed, 7.3% refused to participate, 6.0% could not be located, and 0.8% were unable to participate. Thirty-one percent of the population was born outside South Africa. In Table 1 we report the baseline mean values of the biological and clinical risk factors by sex. The cohort was 46.4% male, had a mean age of 61.7 (±13.1) years, and women had both a higher mean weight (72.7 ± 19.1 kg; *p* < 0.001) and mean systolic blood pressure (138.1 ± 23.3 mmHg; *p* = 0.703) compared to men (70.0 ± 16.9 kg; 137.9 ± 23.5 mmHg). The waist-to-hip ratio (WHR) for both genders was high [19] (0.9 ± 0.1 men; 0.9 ± 0.1 women; *p* < 0.001) and smoking prevalence was substantially higher among men (19.2% vs. 0.4%; *p* < 0.001) (Table 1). DBS tests for HIV antibody tests revealed that 22.5% of the cohort was HIV^+^ (22.4% males; 22.6% females) and among those who tested positive, the presence of ART medications was detected in 662/1,035 (64%).

**Table 1:** Cohort characteristics, Agincourt sub-district, South Africa 2015

Indicator	All			Male			Female			*p-value: Overall*
	*n*	Mean (±SD)		*n*	Mean (±SD)		*n*	Mean (±SD)		
Age (years)	5059	61.7 (±13.1)		2345	61.7 (±12.8)		2714	61.7 (±13.3)		0.990
Weight (kg)	4786	71.5 (±18.2)		2211	70.0 (±16.9)		2575	72.7 (±19.1)		<0.001
Height (m)	4685	1.6 (±10.1)		2160	1.7 (±9.3)		2525	1.6 (±8.2)		<0.001
Average SBP (mmHg)	4895	138.0 (±23.3)		2260	137.9 (±23.5)		2635	138.1 (±23.3)		0.703
Average DBP (mmHg)	4895	82.1 (±12.7)		2260	81.9 (±13.2)		2635	82.3 (±12.3)		0.305
Body Mass Index (kg/m^2)^	4680	27.5 (±10.0)		2156	25.1 (±9.2)		2524	29.6 (±10.2)		<0.001
Waist Circumference (cm)	4758	92.4 (±15.1)		2204	89.4 (±13.3)		2554	95.0 (±16.0)		<0.001
Waist-Hip Ratio	4728	0.91 (±0.08)		2194	0.92 (±0..08)		2534	0.89 (±0..08)		<0.001
Total Cholesterol (mmol/L)	4196	4.24 (±1.3)		1886	4.0 (±1.2)		2310	4.4 (±1.3)		<0.001
High Density Lipoprotein (mmol/L)	4234	1.6 (±0.6)		1911	1.6 (±0.6)		2323	1.6 (±0.5)		0.030
Triglycerides (mmol/L)	4223	1.8 (±1.6)		1903	1.7 (±2.1)		2320	1.8 (±1.0)		0.298
Low Density Lipoprotein (mmol/L)	3841	2.1 (±1.5)		1714	2.0 (±1.9)		2127	2.2 (±1.0)		<0.001
	*n*	Mean (IQR)		*n*	Mean (IQR)		*n*	Mean (IQR)		
Glucose^a, b^ (mmol/L)	4626	6.7 (5.1; 7.2)		2130	6.5 (5.0; 7.1)		2496	6.8 (5.2; 7.2)		<0.001
hsCRP^b, c^ (mg/L)	4296	3.3 (1.2; 4.3)		1955	3.1 (1.1; 4.1)		2341	3.3 (1.4; 4.5)		<0.001
	*n*	Proportion (%)		*n*	Proportion (%)		*n*	Proportion (%)		*p-value: Overall*
Current Smokers	5059	9.1	---	2345	19.2	---	2714	0.4	---	<0.001
HIV Positive^d^	4576	22.9	---	2099	23.0	---	2477	22.8	---	0.872

^a^Point-of-care (random) glucose; ^b^Skewed distributions were log-transformed and the inter-quartile ranges 1(25%) and 3 (75%) are reported; ^c^hsCRP = high-sensitivity C-Reactive Protein; ^d^Self-report and lab assay

In Table 2 we report the prevalence of cardiometabolic disease risk factors based on standard criteria. The prevalence of hypertension was 58% overall with a higher prevalence among women (61.8 vs. 54.5%; *p* < 0.001) as well as an expected increase in prevalence with age. Rates of any dyslipidemia were also high with over 43% of the population having at least one abnormality in their lipid profile. The results of the individual lipid parameters however showed differences between men and women that reflect different risk profiles. Women overall had a higher prevalence of high total and LDL cholesterol compared to men; but lower prevalence of low HDL (31.3% of men; 22.5% of women; *p* < 0.001). The prevalence of diabetes overall was approximately 11%, also increasing with age and with higher rates among women (11.9 vs. 10.1% in men; *p* = 0.048). This measure may be an underestimate given that the prevalence of a blood glucose value greater than 7.0 mmol/L among fasting samples (completed in only one tenth of all participants) was 13.29%. Nearly two-thirds of the cohort had an abnormal BMI with 6.7% being underweight and 58.0% being overweight or obese (44% of men; 70% of women). Nearly 40% of the population had hsCRP levels that are considered high with females having a greater value than men (41.0 vs 35.4% respectively, *p* <0.001).

**Table 2:** Prevalence (%) of risk factors, by sex and age group

	ALL	Male						Female						
		Total Male	40–44	45–54	55–64	65+	*p*-value trend (Male)	Total Female	40–44	45–54	55–64	65+	*p*-value trend (Female)	*p*-value (Overall Men vs women)
Hypertension^a^	58.4	54.5	34.6	42.2	56.9	64.8	<0.001*	61.8	34.6	52.9	65.3	72.0	<0.001*	<0.001
Total Diabetes^b^	11.1	10.1	4.6	8.2	10.1	12.6	0.001*	11.9	4.7	8.7	12.8	15.3	<0.001*	0.048
Self-Report Diabetes	6.7	6.2	1.7	4.5	5.9	8.6	<0.001*	7.1	1.8	4.5	8.3	9.3	<0.001*	0.227
RBG ≥ 11.1 mmol/L	5.5	4.7	1.4	3.9	5.2	5.5	0.044*	6.3	2.4	4.6	5.9	8.5	<0.001*	0.017
Reports diabetes medication use	4.9	4.5	1.7	3.3	4.4	5.9	0.013*	5.3	1.1	3.3	6.6	6.8	<0.001*	0.194
Total Dyslipidemia^c^	43.6	44.8	45.2	50.5	47.5	40.2	0.003*	42.7	41.6	39.8	49.1	40.3	0.002*	0.160
High Total Cholesterol ≥ 6.21 mmol/L	6.6	4.12	3.3	2.9	3.9	5.2	0.291	8.5	4.7	5.9	11.0	9.3	0.002*	<0.001
Low HDL < 1.19 mmol/L	26.5	31.3	35.0	38.4	30.7	27.5	0.001*	22.5	28.3	22.7	24.6	19.2	0.009*	<0.001
High LDL > 4.1 mmol/L	3.7	2.1	2.5	1.2	1.9	2.6	0.458	4.9	3.2	3.9	6.5	5.0	0.136	<0.001
Hypertriglyceridemia > 2.25 mmol/L	21.2	20.4	17.9	23.1	22.3	18.4	0.136	21.7	16.5	18.5	26.0	22.2	0.003*	0.311
BMI [45]														
Underweight: < 18.5	6.7	10.1	10.0	10.9	10.6	9.2	---	3.7	2.3	2.5	4.0	4.6	---	<0.001
Normal: 18.5–24.9	35.4	45.9	51.3	45.0	45.4	45.3	---	26.4	26.3	24.4	21.8	31.1	---	<0.001
Overweight: 25.0–29.9	27.9	27.8	29.1	26.1	26.7	29.2	---	28.1	28.6	26.7	28.8	28.5	---	0.790
Obese Grade I: 30.0–34.9	17.7	11.8	6.9	12.6	12.8	12.1	---	22.7	23.7	23.0	25.7	20.0	---	<0.001
Obese Grade II: 35.0–39.9	7.4	2.8	2.2	3.0	2.9	2.9	---	11.3	12.6	12.9	10.4	10.5	---	<0.001
Extreme Obesity: ≥ 40	4.9	1.5	0.4	2.4	1.6	1.3	---	7.8	5.9	10.6	9.3	5.3	---	<0.001
Waist-Hip Ratio [46]	67.9	61.6	40.9	55.8	66.7	66.7	<0.001*	73.4	62.3	71.2	75.6	76.3	<0.001*	<0.001
hsCRP Risk Category [25]														
Low (<1 mg/L)	19.2	21.7	23.4	22.9	21.3	20.9	---	17.0	16.2	19.24=	15.6	16.9	---	<0.001
Intermediate (1–3 mg/L)	42.4	42.9	43.2	44.6	43.7	41.6	---	42.0	47.0	38.7	42.6	42.4	---	0.517
High (>3 mg/L)	38.4	35.4	33.3	32.5	35.0	37.5	---	41.0	36.8	42.1	41.8	40.7	---	<0.001

^a^Systolic Blood Pressure ≥ 140 mmHg *or* Diastolic Blood Pressure ≥ 90 mmHg *or* Self-reported use of medication.

^b^Self-report *or* Random Blood Glucose (RBG) ≥ 11.1 mmol/L *or* Self-reported use of medication.

^c^Total cholesterol ≥ 6.21 mmol/L *or* low-density lipoprotein (LDL) > 4.1 mmol/L *or* high-density lipoprotein (HDL) < 1.19 mmol/L *or* Triglycerides > 2.25 mmol/L *or* Self-reported use of medication (*n* = 8).

*Statistically significant at α = 0.05

The prevalence of reported advanced cardiovascular conditions such as angina, stroke, “heart attack”, and heart failure was relatively low given the prevalence of risk factors (Table 3). Except for myocardial infarction (MI), women had higher self-reported prevalence of CVD conditions, with a statistically significantly higher (*p* = 0.014) angina prevalence (women 2.84%; men 1.79%), which increased to 8.19% and 12.47% when Rose criteria were included (Table 4). Furthermore, using Rose Criteria [32], we observed a statistically significant increase in the prevalence of angina across age categories for both men (*p* < 0.001) and women (*p* = 0.002). In a multivariate regression of the outcome of angina, the only associations that were significant were age and female sex, after controlling for hsCRP, smoking, Total and HDL cholesterol and blood pressure.

**Table 3:** Self-reported prevalence of CVD conditions

Condition	TOTAL (%)	*p*-value
Angina	2.35	
Male	1.8	0.014*
Female	2.8	
Stroke	3.0	
Male	2.7	0.397
Female	3.1	
MI^a^	0.4	
Male	0.5	0.730
Female	0.4	
Heart Failure	0.8	
Male	0.6	0.102
Female	1.0	

^a^Myocardial infarction

*Statistically significant at α = 0.05

**Table 4:** Angina prevalence by sex and age group

ANGINA	All	Male					All	Female				
Age Group		40–44	45–54	55–64	≥65	*p*-value		40–44	45–54	55–64	≥65	*p*-value
Self-Report or Rose Criteria[32]	8.20	4.1	4.7	8.4	11.1	<0.001*	12.5	7.6	11.4	14.3	13.2	0.024*
Self-Report	1.8	1.7	0.9	1.1	2.8	0.023*	2.8	2.9	2.8	2.4	3.1	0.829
Rose Criteria	6.9	3.3	3.7	7.9	9.0	<0.001*	10.8	5.4	9.3	13.4	11.7	0.002*

*Statistically significant at α = 0.05

The predicted risk of CVD-related events and mortality increased across age categories for both men and women, regardless of the risk scores used; with overall risk notably higher for men than women (Table 5). Over a period of ten years, the Harvard Risk Score predicted a mean absolute risk of cardiovascular death at 11.3% for the entire cohort, while the Framingham score predicted a 12.2% risk for CMD and related mortality, compared to a risk of 9.9% for CMD and related mortality using the ASCVD risk score

**Table 5:** 10-year CVD risk score comparisons by sex and age group^a^

Age	Number (N)	Harvard Risk Score [29] (%)	Pooled Cohort Risk Equations (ASCVD) [27] (%)	Framingham 2008 [28] (%)
WOMEN				
Aggregate	2239	9.6	7.7	9.7
40–44	273	2.3	1.3	3.9
45–54	667	5.2	3.6	7.1
55–64	708	10.0	7.0	10.9
65–74	591	17.6	15.0	13.8
MEN				
Aggregate	1964	13.4	12.5	15.3
40–44	241	3.8	4.8	5.8
45–54	508	7.3	7.6	9.6
55–64	659	14.2	13.1	16.5
65–74	556	22.0	18.3	21.6
TOTAL				
Aggregate	4203	11.3	9.9	12.2
40–44	514	3.0	2.8	4.7
45–54	1175	6.1	5.2	8.1
55–64	1367	12.0	10.4	13.5
65–74	1147	19.7	16.6	17.6

^a^Excludes persons ≥ 75 years or BMI < 7 or BMI > 100 kg/m^2^ or DBP > 120 mmHg.

### HIV status

Evaluation of CMD risk factors by HIV status revealed an increased risk for cardiovascular disease. The mean age for those who are HIV^−^ was 63 years, compared to 55 years for those who are HIV^+^. Furthermore, the prevalence of hypertension, diabetes, and obesity was significantly higher among those without HIV infection (Table 6). Additionally, HIV^+^ males had a significantly higher prevalence of underweight (*p* = 0.007) compared to their HIV^−^ counterparts (Table 6) and higher hsCRP levels (3.68 vs 3.12, *p* < 0.001; data not shown). Furthermore those without HIV-infection had consistently higher proportions of multiple CMD co-morbidities, compared to their HIV^+^ counterparts. For example, nearly 35% of HIV^+^ persons had 2 or more conditions compared to nearly 51% among HIV^−^ persons (Table 7). Women also had a consistently higher prevalence than men across all categories, regardless of HIV-infection status (Table 7). When including HIV-infection as a co-morbid condition, overall, 55.7% (*n* = 2,820) of the cohort had at least 2 co-morbid CMD conditions (hypertension, diabetes, dyslipidemia, angina, obesity, heart failure, HIV^+^), 24.5% (*n* = 1,239) had at least 3, and 6.8% (*n* = 344) had at least 4 (Table 7). Risk of multiple co-morbidities also generally increased with age, as expected (data not shown).

**Table 6:** Prevalence (%) of risk factors by HIV status

	Male			Female		
	HIV^−^ (*n = 1186)*	HIV^+^ (*n = 524)*	*p*-value	HIV^−^ (*n = 2093)*	HIV^+^ (*n = 610)*	*p*-value
Hypertension^a^	59.2	38.7	<0.001*	67.2	43.8	<0.001*
Diabetes^b^	10.9	7.3	0.014*	13.1	7.9	<0.001*
Dyslipidemia^c^	45.4	42.7	0.303	42.8	42.2	0.810
BMI (kg/m^2^)[45]						
Underweight (<18.5)	9.1	13.3	0.007*	3.5	4.5	0.241
Normal (18.5–24.9)	44.3	51.5	0.005*	23.5	35.2	<0.001*
Overweight (25.0–29.9)	29.3	22.7	0.004*	28.7	26.2	0.225
Obese Grade I (30.0–34.9)	12.9	8.6	0.010*	23.9	18.6	0.007*
Obese Grade II (35.0–39.9)	3.3	1.6	0.070	11.7	10.2	0.321
Extreme/Morbid (>40)	1.3	2.3	0.142	8.6	5.2	0.007

*Note*: HIV status is defined as self-report or positive on assay analysis.

^a^Hypertension defined as SBP > 140 mmHg or DBP > 90 mmHg or Reports currents use of anti-hypertensive medication;

^b^Diabetes defined as Self-Report or Fasting Blood Glucose >7.0 mmol/L or Random Blood Glucose > 11.1 mmol/L;

^c^Dyslipidemia defined as elevated total cholesterol (>6.21 mmol/L) or high LDL (>4.1 mmol/L) or Low HDL (<1.19 mmol/L) or Hypertriglyceridemia (>2.25 mmol/L);

* Statistically significant at α = 0.05

**Table 7:** Prevalence of Select Co-morbidities^a^ by Sex and HIV Status^b^

Number of Co-Morbidities^a^	≥2 (%) *n* = 2,820	≥3 (%) *n* = 1,239	≥4 (%) *n* = 344
HIV^−^	50.5	21.6	5.3
Male	41.9	15.8	2.9
Female	57.9	26.6	7.4
HIV^+^	34.6	12.0	3.3
Male	28.2	9.0	2.5
Female	40.0	14.6	3.9

^a^Comorbidities are clinically measured hypertension, diabetes, hyperlipidemia, angina (Rose criteria only), BMI > 30, HIV status and self-reported history of heart failure, myocardial infarction, and stroke.

^b^HIV status is defined as self-report and positive on assay analysis.

## Discussion

Our baseline findings from the HAALSI study indicate that CMD risk is high in this rural, middle-aged and older South African cohort and indeed, when coupled with the high prevalence of HIV, it is evident the population is experiencing a complex epidemiologic transition characterized, in part, by an intense and interacting double burden of cardiometabolic and infectious disease. The cohort is at increased risk for CMD based on the distribution of traditional risk factors some of which fall at, or beyond, the upper limit for the rural or provincial prevalence reported for the province in a nationally representative sample [17]. Approximately 80% of both men and women had hsCRP levels in the intermediate to high range which likely reflects elevated systemic inflammation (Table 2). While this could involve a number of factors, the high level of overweight and obesity recorded suggests that body fat distribution may be a key influence. The increase in hsCRP needs further evaluation to assess the magnitude of risk and to determine if it serves as a greater predictor of risk in this population [6, 16, 35].

The prevalence of hypertension, diabetes and obesity are greater among women than men, however women had lower rates of low HDL-levels and smoking. The greater obesity in women is consistent with other studies in South Africa and the region [17, 36]. Smoking rates overall are relatively low and men report current smoking at almost 23 times the rate of women. Despite this disparity between genders, smoking is likely under reported for both groups as the SANHANES Study reports that 55-79% of men and 53-62% of women report smoking daily. While the overall lipid profiles for the cohort were comparable to participants in the SANHANES Study [17], men and women in HAALSI had higher HDL levels than those measured for SANHANES participants older than 35. Still, the increased risk of CVD due to abnormal HDL levels continues to be of concern in this predominantly Black population since the SANHANES sample measured the highest levels of abnormal HDL in rural populations, with 1 out of 2 black men and women aged 15–65 having this risk factor. The WHR of women in our cohort was also higher (0.89 vs. 0.85), as it was for men (0.92 vs. 0.87), compared to SANHANES.

Additionally, the burden of HIV-infection adds to the prevalence of those with significant and multiple co-morbidities, corroborating findings from a previous study in the Agincourt population where more than 50% had at least one chronic condition (CVD or HIV). In that study, CMD risk factors increased with age except for low levels of HDL [14]. The biggest drivers of CMD in the present cohort are abdominal obesity and hypertension, and also dyslipidemia and smoking for men. SANHANES, further emphasizing the cohort’s increased CMD risk.

The United Nations estimated that approximately 5 million men and women over the age of 15 were living with HIV in South Africa in 2014 [37]. With ART coverage now so extensive, it is likely that the cohort population will benefit from an extended lifespan. As a result of this increased longevity, we would expect an increase in the prevalence of CMD and associated risk factors among those who are HIV^+^. In this cohort we observed that the dyslipidemia prevalence is similar amongst HIV^+^ and HIV^−^ persons, which is likely due to the higher prevalence of obesity in the former population.

The prevalence of hypertension in the overall cohort is at the high end of the observed range in the SANHANES sample which may be explained, at least in part, by the high obesity levels. The impact of HIV-infection on the overall prevalence however has competing influences. HIV^+^ status has been associated with lower BMI in many African populations [38], which can lower blood pressure. In contrast high levels of inflammation in the general ageing population has been shown to be associated with increases in blood pressure [39]. It is unclear what the contribution of elevated hsCRP is towards developing hypertension in this HIV^+^ population with varying levels of ART. The overall risk of future co-morbid and fatal events appears to be considerable in this population. Follow-up mortality and cause-of-death data on cohort participants, derived from the HDSS, will allow us to define more sharply the upcoming CMD burden and associated mortality expected in this cohort and, indeed, throughout much of rural South Africa where a similar risk and morbidity profile pertains [17].

This initial study serves as a valuable assessment of CMD conditions in this rural aging population where the prevalence of many of the conditions was found to be quite high. More importantly, it sets up a baseline to compare in the ensuing planned longitudinal follow-up. In the follow-up study, we will be able to evaluate the rate of change of the CMD conditions in this population. While the younger age of the HIV^+^ cohort may explain some of the lower prevalence of CMD conditions, other factors such as increasing use of ART may change this. We also will be able to assess the impact of treatment of HIV-infection on the change in prevalence of CMD conditions, and improved or diminished access to care. Further we plan to specifically focus on the impact of inflammatory markers on the progression of vascular disease. Finally, we will be able to look at the impact of changes in migratory status on health and health system seeking behaviors. Further analyses will include evaluating electrocardiograms and echocardiograms to see if there is confirmatory evidence of underreporting of CMD conditions.

### Strengths

Earlier findings show that temporary (largely labor) migrants returning to Agincourt after periods in towns carry a higher risk of death from noncommunicable diseases (NCDs), thus identifying the internal migrants as a risk group warranting further investigation [40, 41]. A major strength of this study is that the Agincourt HDSS is well prepared to undertake follow-up of residents in the catchment area of the study, and also to track internal migrants who move temporarily or permanently out of the sub-district. The large sample size, the wide range of cardiovascular disease-related variables collected, and the collection of biological samples are all added strengths.

### Limitations

This data has limitations associated with all cross-sectional assessments, limiting the extent to which causal inferences can be made. The use of point-of-care (POC) testing for lipid and glucose levels is also a potential limitation, when compared to measurements obtained through conventional laboratory testing. However, the machines used to obtain POC testing have been validated against current laboratory-based methods [42, 43].

## Conclusions

This cohort is experiencing an increased risk of CMD with age, as expected. Further, it appears that the HIV^+^ population has many of the chronic CMD risk factors to contend with as well. With the increase in co-morbidities associated with HIV-infection, their longer-term impact on screening, prevention, and treatment of CMD needs to be better understood. Indeed, the health system challenges posed by the evolving chronic disease trajectories are profound with little precedent elsewhere.

The establishment of the HAALSI cohort is timely in order to assess the evolution of long-term CMD risks in a rural population that is living longer with HIV (and is thus exposed to prolonged antiretroviral therapy); and to inform the many parts of South and sub-Saharan Africa confronting similar realities. The INDEPTH Network of population-based research centers – reflecting the range of transitional settings across southern, East and West Africa – provides a potential vehicle for harmonized research to generate the evidence essential to policy and practice [44].

## Abbreviations

AIDS: Acquired immune deficiency syndrome
ART: Anti-retroviral therapy
ASCVD: Atherosclerotic cardiovascular disease pooled risk score Equations
BMI: Body mass index
CAPI: Computer assisted personal interview
CMD: Cardiometabolic diseases
CRP: C-reactive protein
DBS: Dried blood spots
FBG: Fasting blood glucose
HAALSI: Health and Aging in Africa: Longitudinal Studies of INDEPTH Communities
HDL: High-density lipoprotein
HDSS: Health and socio-demographic surveillance system
HIV: Human immunodeficiency virus
hsCRP: High-sensitivity C-reactive protein
LDL: Low-density lipoprotein
MI: Myocardial infarction
NCDs: Noncommunicable diseases
PLWHA: People living with HIV/AIDS
POC: Point-of-care
RBG: Random blood glucose
SANHANES: South African National Health and Nutrition Examination Survey
SSA: Sub-Saharan Africa
WHO: World Health Organization
WHR: Waist-to-hip ratios
